# Effects of privacy concerns on older adults’ discontinuous usage intention: the chain mediating effect of technology anxiety and the moderating role of perceived price value

**DOI:** 10.3389/fpubh.2025.1543409

**Published:** 2025-05-13

**Authors:** Jing An, Kexin Wan, Ziyue Xiang, Xuanyu Zhu, Jinlong An, Yujie Yang

**Affiliations:** ^1^School of Management, Nanjing University of Posts and Telecommunications, Nanjing, Jiangsu, China; ^2^The Fourth People’s Hospital of Shenzhen, Shenzhen, Guangdong, China; ^3^First People’s Hospital of Changshu City, Hospital Affiliated to Soochow University, Changshu, Jiangsu, China

**Keywords:** privacy concerns, discontinuous usage intention, technology anxiety, perceived price value, dissatisfaction, mediating effect, moderating role, older adults

## Abstract

**Introduction:**

With the continuous progress of global information and communication technology, online medical care has become a hot topic. And online health consultation platforms have also emerged and provided patients, especially the older adults, with a new way to obtain health information.

**Objective:**

To explore the impact of privacy concerns, technology anxiety, dissatisfaction, and perceived price value on older adults’ discontinuous usage intention of online health consultation platforms.

**Methods:**

Using quantitative analysis method, 254 valid questionnaires were collected online with the sample of older adults aged over 50 years in China. Descriptive statistics and structural equation models were established, and hypothesis testing was carried out using SPSS software.

**Results:**

In this study, privacy concerns (*β* = 0.268, *p* < 0.001), technology anxiety (*β* = 0.256, *p* < 0.001), and dissatisfaction (*β* = 0.433, *p* < 0.001) directly affect the discontinuous usage intention of online health consultation platforms, among which technology anxiety and dissatisfaction play a mediating role (*p* < 0.01). Price value moderates the impact of privacy concerns on discontinuous usage intention. The results of this study indicate that the mental state of older adults using the online health consultation platform will significantly affect their dissatisfaction and discontinuous usage intention of the platform.

## Introduction

With the continuous progress of information communication technology (ICT), online health consultation platforms have become a source of access to medical health information for patients, especially during the spread of large epidemics. Online health counseling provides a way for patients to obtain social support and professional guidance from doctors and psychological encouragement from users. Online health counseling has many advantages over traditional face-to-face medical situations, without time and place constraints (1). Patients do not need to go to offline hospitals or clinics but only need to connect to the Internet to get professional medical guidance and disease treatment help (2). Anonymous consultation is a unique form of online consultation, which makes patients more willing to expose health-related privacy during medical consultation. There is some situation that patients may conceal during offline medical treatment, which may affect doctors’ judgment of the patient’s real condition (3). Therefore, online health consultation has gradually become a new mode of “doctor-patient communication.”

Health consultation refers to the provision of consulting services for health issues by using the techniques and methods of health consultation. Online paid health consultation platforms offer remote medical consultation services, allowing users to pay fees and receive professional advice from doctors through internet media. In China, online health consultation platforms represented by JD Health primarily offer services through mobile applications. These platforms mainly use a combination of text and images along with AI-assisted consultation methods, and also provide the option of video consultations. The doctors on these platforms are mostly part-time staff from public medical institutions. In the United States, online medical platforms like Teladoc Health support both web and mobile access, with video consultations being the primary mode of service, and also offer telephone consulting services. The doctors on these platforms are typically full-time online workers, and some collaborate with offline clinics such as CVS Health. In Europe, although online medical platforms also mainly provide services through websites and applications, their service models combine elements of both online and offline services, not relying entirely on online consultations. European users tend to prefer the public healthcare system and have a relatively lower acceptance of paid consultations, although platforms like Kry are popular among younger people. In conclusion, whether it is an online paid health consultation platform from any country or region, users can obtain health information through paid consultation. Currently, due to the greater degree of proficiency and adoption of information technology among younger individuals, the primary user base for online paid health consultation platforms is also the younger group.

Due to the lack of age-friendly interfaces or the integration of assistance features on these platforms, older adults face numerous difficulties when using them, resulting in relatively low participation rates. The “digital divide” not only hinders their online social interactions but also generates negative emotions toward technology use, leading to a lack of trust in the services provided by online healthcare. The use of Internet may lead to the exposure of user privacy, a point that has been acknowledged by the majority. However, young people with higher information literacy tend to be more confident in avoiding the risks of privacy exposure, whereas older adults may worry that their unfamiliarity with information technology might inadvertently lead to the disclosure of personal information. When older adults use online information technology, especially those online health consultation services that require them to provide personal data, they often feel concerned about privacy issues, which is referred to as privacy concerns. At the same time, older adults may also fear about the potential issues that may arise from using emerging technologies, a phenomenon commonly referred to as technology anxiety. The imbalance between the literacy level of older adults and the services they hope to obtain through technology often exacerbates this negative emotion. Furthermore, when older adults use online paid health consultation platforms, they need to pay a certain fee to access the services. Whether the quality of the service matches the price will affect their decision to continue using these consultation service platforms. Therefore, the role of price value cannot be ignored.

We construct the research model to measure older adults’ discontinuous usage intention of online paid health consultation platforms based on the Technology Acceptance Model (TAM), and incorporate the use characteristics of older adults by introducing two variables, technology anxiety and privacy concerns, to propose a new theoretical model to understand the factors influencing older adults’ discontinuous usage intention of online paid health advice platforms. TAM is widely applied in information systems research, and the model mainly includes variables such as perceived ease of use and satisfaction in measuring the use of technology. Subsequent research has expanded it by incorporating external factors such as social influence and facilitating conditions, which has enhanced the explanatory power of the model. Our research model selects the dissatisfaction and discontinuous usage intention variables from the TAM model. Previous studies rarely simultaneously experimented with technology anxiety and privacy concerns in a research model. This study not only considers both factors, but also measures their influence on discontinuous usage intention among older adults. In addition, we consider the characteristics of older adults’ using online paid health advice platforms, and use price value as a moderating variable to analyze the sensitivity of older adults population to price value. Our study not only enriches research on older adults’ discontinuous usage intention of online paid health consultation platforms, but also contributes to the research on the relationship between technology anxiety and privacy concerns.

This study aims to analyze the factors that influence older adults’ discontinuous usage intention of online paid health consultation platforms and discuss how to optimize online paid health consultation platforms to play a role in improving the use of older adults, so that older adults can better enjoy the fruits of the rapid development of technology.

## Literature review

In the literature studying online health counseling, there is very limited research on individual adoption of online health counseling services. Some studies explored influencing factors of health information seeking for online health services (OHS, Online Health Service). Trust, perceived risk, perceived usefulness, and perceived health status were common factors influencing consumer information-seeking behavior (4). Obviously, information-seeking and online counseling are related but different behaviors. Information seeking is usually one-sided, requiring only individual users using the web to search for pre-existing health information, while online consultation involves interaction between the patient or the patient’s family and the doctor and requires bipartite effort. Some studies discussed the behavior of online health service use in a comprehensive situation, including information search, online registration, online consultation, and patient education, rather than just the single behavior of information search (5, 6). From the perspective of research objects, most studies focus on middle-aged or general groups with a high frequency of Internet use. There are few studies on older adults. Older adults have much demand for health information consulting, which can help them to answer their health questions. However, for the majority of older adults, the advanced information technology use experience is limited (e.g., smartphone), and their physical health is poor (such as weak eyesight). As a vulnerable group with digital intelligence ability, older adults groups need to be paid attention to by the government, technology developers, and service providers.

A summary of the existing literature with author information, influencing factors, and main findings is shown in Table 1.

**Table 1:** Literature summary.

References	Conclusion	Influencing factor
Lim et al. (35)	Technology anxiety is not related to willingness to use in the application environment	Technology anxiety
Xue et al. (9)	Neither technology anxiety nor perceived physical condition is directly related to perceived ease of use and usefulness, lifestyle and intention to use.	Technology anxiety
Deng et al. (8)	Perceived value, attitude, perceived behavioral control, technology anxiety, and need for self-actualization positively influenced the behavioral intentions of older users; technology anxiety had a significant effect on the behavioral intentions of the older group, but not on the middle-aged group.	Technology anxiety
Hoque and Sorwar (36)	Performance expectations, effort expectations, social influence, technology anxiety and resistance to change have a significant effect on users’ behavioral intentions to adopt mHealth services. Technology anxiety has a significant but negative effect on intention to use mHealth.	Technology anxiety
Khan et al. (24)	There is no negative correlation between technology anxiety and mHealth adoption intentions.	Technology anxiety
Meng et al. (41)	Technology anxiety enhanced the effect of affective trust, but not the effect of cognitive trust on continued willingness.	Technology anxiety (modulating effect)
Kim et al. (10)	Lower levels of digital literacy among older adults may limit their access to health information and negatively impact their health. Differences in technology use anxiety between males and females were statistically significant, with males having higher potential means than females.	Technology anxiety
Stewart & Segars (42)	Concern for information privacy moderates the relationship between computer anxiety and behavioral intentions.	Technology anxiety & Privacy concern
Czaja et al. (7)	Computer anxiety, fluid intelligence, and crystallized intelligence were significant predictors of technology use. The relationship between age and technology adoption was influenced by cognitive ability, computer self-efficacy and computer anxiety.	Technology anxiety
Bansal et al. (20)	Individuals’ intentions to disclose such information depend on their trust, privacy concerns, and information sensitivity, which are determined by personal predispositions (personality traits, information sensitivity, health status, prior privacy invasions, risk beliefs and experiences).	Privacy concern
Zhou and Li (43)	The three factors of subjective norms, social identity and group norms and privacy concerns had a significant effect on continued use. Privacy concerns and privacy risks have a negative effect on continued use.	Privacy concern
Osatuyi (22)	Information privacy concerns as a mediator influencing the relationship between computer anxiety and behavioral intentions.	Technology anxiety and privacy concern
Bansal et al. (37)	Contextual sensitivity influences off moderating the effects of personality and personal experience on privacy issues and trust.	Privacy disclosure
Church et al. (44)	Older people’s lack of privacy awareness and insufficient knowledge of information technology to protect their privacy.	Privacy concern
Fox and Connolly (13)	Health information privacy concerns are negatively associated with mHealth adoption intentions.	Privacy concern
Fox et al. (21)	Individuals’ perceptions of both reciprocity and health benefits associated with apps positively influence willingness to rely, while privacy concerns have a negative but weak effect.	Privacy concern
Pool et al. (45)	Privacy practices (e.g., informed consent) can help reduce older adults’ concerns and increase their acceptance of telemedicine.	Privacy concern
Zhang and Zhang (17)	There is a significant relationship between technology anxiety and technology, provider, legal, and user vulnerabilities, but technology anxiety does not have an impact on privacy issues.	Privacy concern and technology anxiety
Venkatesh et al. (14)	The effect of hedonic motivation on behavioral intentions is stronger for younger men with less technical experience, while the effect of price value is more important for older women.	Price value
Alalwan et al. (15)	Performance Expectation, Effort Expectation, Hedonic Motivation, Price Value and Trust have significant positive effects on behavioral Intention.	Price value
Alam et al. (16)	The effect of price value on behavioral intention to adopt mHealth was weak and insignificant.	Price value

It can be seen that there are few studies on factors influencing the use of information technology in which older people have been selected as research subjects. Some studies indicated that older people are less capable of using Internet information technology (7). Technology anxiety, as negative emotion, occurred in older people’s use of information technology, but the role of this factor in influencing older people’s willingness to use it in an unsustainable manner was not conclusively established. A study conducted in China (8) indicated that older adults’ emotion of technology anxiety directly affected their use of mHealth services. Whereas a study conducted in Singapore (9) indicated that technology anxiety among female older adults did not affect their use of mobile phones for health information. In addition, the acceptability of smart devices among Korean older adults was investigated (10). There were differences in e-health literacy and technology use anxiety between males and females. Privacy concerns, as another negative emotion that may arise when using technology, may be more severe among older adults (11). Older adults do not pay attention to the privacy options of e-products or services due to their lack of privacy awareness and insufficient IT knowledge and may unconsciously disclose their personal information and fail to protect their privacy on their own (12). It is shown that privacy concerns among older adults affect their use of mobile healthcare technology (13). Most of previous research on price value has been used in marketing studies. Some studies used price-value as direct influence on use and suggested that this variable has an impact on willingness to use (14–16).

This study focuses on online paid health advice platforms discontinuous usage intention among older adults, attempting to examine discontinuous usage intention by looking at the negative affective aspects of older adults in conjunction with their technology use characteristics. Technology anxiety and privacy concerns are rarely simultaneously considered in a research model. Price value, as one attribute of online paid counseling platforms, was innovatively considered as a moderating variable in our study, indicating the novelty in terms of model construction and a supplement to existing research.

## Model and hypothesis development

### Model variables

Based on previous literature and the objectives of this study, the study will construct a theoretical model grounded in the TAM, incorporating the psychological characteristics of older adults’ technology use—such as privacy concerns, technology anxiety, dissatisfaction, and discontinuous usage intention—along with external variables related to the features of online paid health consultation platforms, specifically price value.

### Privacy concerns

The use of AI, cloud computing services, and big data provides accurate aging transformation, which not only requires the form of interaction, but also fully considers the internal needs and use experience of older adults users. Scholars believe that personal concerns about health data privacy can affect their willingness to use the technology (17–19) and their physical and mental health (20). The privacy concerns generated by older people when using IT platforms may trigger dissatisfaction and unwillingness to use them. Therefore, the following hypotheses are proposed:

*H1*: The privacy concerns arising from use of online paid health consultation platforms have a positive impact on discontinuous usage intention of older adults.

*H2*: The privacy concerns arising from use of online paid health consultation platforms have a positive impact on older adults’ dissatisfaction.

With the increasing degree of aging adaptation, individual privacy data is collected more comprehensively, and the risk of privacy invasion is rising. The issue of privacy protection under intelligent information products has also been widely discussed by scholars. It has been shown that privacy concerns hurt the willingness to adopt APP and the willingness to rely on APP (21). The anxiety of users about using information technology is also influenced by their technical capabilities (22). As older adults’ group whose ability to use technology is lower than the normal level, they are afraid of invading their privacy and are more vulnerable to technological anxiety. Therefore, the following hypothesis is proposed:

*H3*: The privacy concerns arising from use of online paid health consultation platforms have a positive impact on technology anxiety among older adults.

### Technology anxiety

Technology anxiety refers to the fear of individuals facing problems when using new technology. Technical anxiety can be a significant feature of older users (23). Aging is a specific process that older adults group is facing. In the process of aging, the sensory and motor system functions of older adults gradually decline, and older adults will encounter more difficulties when using emerging technologies, which will make older adults users feel confused or uneasy in the process of using online consultation services (8). Online health consultation services, as an emerging application of technology, have not yet been widely popularized. Therefore, older adults are not yet familiar with this entirely new technology, and the help provided by society is relatively limited. Because the frequency of older adults using technology platforms is much lower than that of young people, some platforms tend to prioritize meeting user experience needs of the majority of young users. As a result, most of the technical platform are aimed at young groups, providing less technical services to older adults and less consideration for the application of older adults (7, 20). This leads to older adults being more likely to encounter challenges when operating technology platforms, while the development of online health consultation platforms is still in its early stages, and improvements for aging suitability still need to be strengthened. This situation will have a certain impact on the technical acceptance psychology of older adults, resulting in uncomfortable and unsatisfactory negative psychology (24), which will affect the willingness of older adults to continue to use the online paid health consultation platform. Therefore, the following hypotheses are proposed:

*H4*: Technology anxiety arising from use of an online paid health consultation platform has a positive impact on discontinuous usage intention of older adults.

*H5*: Technology anxiety arising from use of an online paid health consultation platform has a positive impact on older adults’ dissatisfaction.

### Dissatisfaction

Expectation disconfirmation theory holds the view that users’ willingness to continue to use depends on satisfaction, which mainly includes three factors, individual initial expectation, perceived performance, and perceived disconfirmation. User satisfaction is the most critical element in the research related to the use of information systems, given the close correlation between satisfaction and the continuous use of information systems. Scholars have proved that satisfaction has the greatest impact on users’ discontinuous usage intention (25), and vice versa, as dissatisfied users are more likely to have the intention not to continue to use (26). With the rise of the economic level, the health status of some to older adults has been somewhat improved. However, older adults also face the economic distress of overburdened medical expenses and mental health disconnection from the rapidly developing technology. Internet use among older adults mainly focuses on social activities (11) and access to information (27), and the use of the Internet had some negative effects on the health of middle-aged and older adults (10). Lack of Internet supervision and information asymmetry may easily cause the dissatisfaction of older adults, and directly or indirectly affect their willingness to continue to use the online paid health consultation platform. Therefore, the following hypothesis is proposed:

*H6*: The dissatisfaction generated by use of the online paid health consultation platform has a positive impact on the discontinuous usage intention of older adults.

### Price value

Price value refers to the trade-off between the perceived utility resulting from using the application and the currency price of the application (28). Studies have shown that the cost and pricing structure may have a significant impact on consumer use of technology (16). Price value plays a positive role when the benefits of using a technology are considered to be greater than the monetary cost, and this price value has a positive effect on intention. As price-sensitive groups, older adults will not only pay attention to the effectiveness of the technical services but also to the rationality of its pricing (14). Therefore, the behavior of older adults using the information technology platform will be affected by the pricing of the platform and its psychological perceived value. Therefore, we take price value as a moderating variable and put forward the following assumptions:

*H7*: Price value moderates the relationship between privacy concerns and discontinuous usage intention.

*H8*: Price value moderates the relationship between privacy concerns and discontinuous usage intention through technology anxiety.

*H9*: Price value moderates the relationship between privacy concerns and discontinuous usage intention through dissatisfaction.

*H10*: Price value moderates the relationship between privacy concerns and discontinuous usage intention through technology anxiety and dissatisfaction.

**Figure 1:**
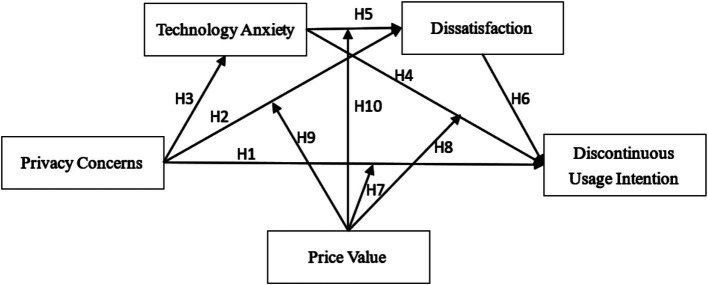
Research model (49).

Combined with the characteristics of online paid health consultation platforms, the model of older adults’ discontinuous usage intention is constructed (as shown in Figure 1). When constructing our research model, we retain the two variables of discontinuous usage intention and dissatisfaction from the TAM model, and add three other variables of technology anxiety, privacy concerns, and price value. We creatively simultaneously included technology anxiety and privacy concerns as the mediating variables in the model, and measured their role in influencing discontinuous usage intention. In summary, the model of this study is a typical chained mediation model, where the independent variable is privacy concerns, the mediating variables are technology anxiety and dissatisfaction, the dependent variable is discontinuous usage intention, and the moderating variable is price value, indicating the novelty of our research model.

## Methods

### Data collection

At present, Chinese society is undergoing a phase of population aging. This study selects individuals aged 50 and above as the research subjects and defines them as older adults. This group is facing increased health risks, such as the emergence of chronic diseases and the decline of physical functions, while their social roles are also changing, such as retirement and changes in family structure. Therefore, conducting research on the health technology usage behavior of this group is of significant practical importance. The website “www.wjx.cn” was used to design online questionnaires, and distributed through the WeChat community filled in by the young family members of older adults. Paper questionnaires were sent to older adults living in the residential area. A total of 300 questionnaires were collected. The data that has been taken less than 1 min to answer the questionnaire or that did not meet the age requirements or data with missing information were excluded. The number of valid samples obtained after the exclusion is 254, which meets the minimum sample size requirements as required in previous studies (29, 30).

### Questionnaire design

To determine the content validity of the model structure, measurement items for all variables were used and adapted from well-established scales from previous studies (see Table 2).

**Table 2:** Scale items.

Variable	Measuring items	Source
Privacy concern	PC1. Using an online paid health consulting platform will result in my losing control of personal information privacy.	Suh and Li (48)
	PC2. Using an online paid health consulting platform can lead to my privacy disclosure because my personal information may be used without my knowledge.	
	PC3. Using an online paid health consulting platform may let others control my personal information.	
Technology anxiety	TA1. Using an online paid health consulting platform will make me very nervous.	Xue et al. (9)
	TA2. Using an online paid health consulting platform may make me uncomfortable.	
	TA3. Using online paid health consulting platforms may be disturbing and confusing to me.	
Dissatisfaction	DS1. I am not satisfied about my overall experience using the online paid health consulting platform.	Zhang et al. (19)
	DS2. I am not pleased about my overall experience using the online paid health consulting platform.	
	DS3. I am not contented about my overall experience using the online paid health consulting platform.	
	DS4. I am not delighted about my overall experience using the online paid health consulting platform.	
Discontinuous usage intention	DUI 1. I will use the online paid health consulting platform less in the future.	
	DUI 2. I will use other forms of health consultation (such as offline physical medical institutions).	
	DUI 3. Sometimes I briefly stop using the online paid health advice platform for a while and then use it again.	
	DUI 4. If I can, I will stop using the online paid health consultation platform.	
Price value	PV1. I can use the services of the online paid health consulting platform at a reasonable price.	Venkatesh et al. (14)
	PV2. Online paid health consulting platform service values for money.	
	PV3. At current prices, the online paid health consulting platform provides good value.	

The structured questionnaire was originally written in English. Based on previous research (31), we used a combination of back-translation, committee and pre-testing methods. During the translation process, two bilingual experts, two bilingual graduate students in information systems, and an English-only expert compared the original English version and the back-translated English version of the questionnaire. This process continued until they found the two English versions to be identical.

The questionnaire was divided into two parts. The first part is the demographic variables. The demographic variables included gender (1 = male, 2 = female), age (1 = 50–54 years, 2 = 55–60 years old, 3 = 61–64 years old, 4 = 65–70 years old, 5 = over 71 years old), marital status (1 = single, 2 = married, 3 = divorced, 4 = widowed), education level (1 = primary school and below, 2 = junior middle school, 3 = senior middle school, 4 = bachelor degree, 5 = master degree, 6 = doctoral degree), family annual income (1 = less than 50,000 yuan, 2 = 50,000–100,000 yuan, 3 = 100,000–200,000 yuan, 4 = more than 200,000 yuan), work associated with information technology/network/computer (1 = very related, 2 = related, 3 = unrelated), current physical condition (1 = healthy, 2 = general, 3 = poor), and resident community (1 = city, 2 = rural areas). The second part is the variables involved in the research model. All items were measured using the Likert 7-point scale (1 = very disagreeable to 7 = very agreeable).

### Procedure

IBM SPSS, AMOS, and Process were used in this study. The structural equation modeling (SEM) was used to validate the research model. The study scale was first tested for both reliability and validity. Descriptive statistical characteristics of all demographic variables were then determined. Finally, we tested the fit of the study model, and conducted hypothesis tests using AMOS and Process, focusing on the mediation effect of technology anxiety and dissatisfaction, and explored the moderating effect of price value.

## Results

### Descriptive statistics

There were 129 male respondents and 125 female respondents. In terms of age distribution, the number of respondents aged from 50 to 54 was 122. The majority of marital status of the respondents was married (*n* = 95). The majority of the respondents had a primary school education and below (*n* = 67). Most of the respondents’ annual income was between 50,000 and 100,000 yuan, accounting for 34.6%. Most of the respondents’ jobs were very related to information technology, network or computer, accounting for 37.8%. The majority of the respondents used the Internet for 1–3 years, accounting for 29.5%. The majority of the respondents were healthy (*n* = 142). Most of the respondents lived in urban communities (*n* = 169), as shown in Table 3.

**Table 3:** Distribution of demographic variables.

Variable	Items	Frequency
Gender	Male	129
	Female	125
Age	50–54 years	122
	55–60 years	54
	61–64 years	39
	65–70 years	15
	Over 71 years	24
Marital status	Single	94
	Married	95
	Divorced	41
	Widowed	24
Education level	Primary school and below	67
	Junior middle school	45
	Senior middle school	50
	Bachelor degree	66
	Master degree	9
	Doctoral degree	17
Family annual income	Less than 50,000 yuan	70
	50,000–100,000 yuan	88
	100,000–200,000 yuan	53
	More than 200,000 yuan	43
Work associated with information technology/network/computer	Very related	96
	Related	66
	Unrelated	92
Network use experience	Less than 1 year	74
	1–3 Years	75
	3–5 Years	37
	More than 5 years	68
Current physical condition	Healthy	142
	General	68
	Poor	44
Resident community	City	169
	Rural areas	85

### Reliability and validity tests

Confirmatory factor analysis was used to assess the validity and reliability of the scale. Anderson and Gerbing (32) proposed three *ad hoc* tests to check convergence validity, including standardized factor load (FL), composite reliability (CR), and mean–variance extraction (AVE). All FLs have values above 0.70, ranging from (0.701) to (0.858), indicating that the model is suitable for SEM (33). In the reliability test, the Kronbach *α* and CR scores for all factors were greater than the expected minimum limit of 0.70. Furthermore, the AVE was above the minimum acceptable point of 0.50 (34). Therefore, the results in Tables 4, 5 indicate that the convergent reliability and validity of the scale are good.

**Table 4:** Reliability and validity test.

Variable	Question item	N	Cronbach’s α	Loadings	AVE	CR
PC	PC1	3	0.867	0.779	0.639	0.779
	PC2			0.888		
	PC3			0.819		
TA	TA1	3	0.842	0.759	0.642	0.843
	TA2			0.848		
	TA3			0.795		
DS	D1	4	0.869	0.808	0.624	0.869
	D2			0.790		
	D3			0.772		
	D4			0.790		
PV	PV1	3	0.864	0.839	0.681	0.865
	PV2			0.835		
	PV3			0.801		
DUI	DUI1	4	0.863	0.827	0.613	0.864
	DUI2			0.768		
	DUI3			0.771		
	DUI4			0.764		

PC, privacy concerns; TA, technology anxiety; DS, dissatisfaction; PV, price value; DUI, discontinuous usage intention.

**Table 5:** Discriminant validity.

Latent variables	DUI	DS	TA	PC	PV
DUI	**0.783**				
DS	0.670**	**0.790**			
TA	0.544**	0.430**	**0.802**		
PC	0.541**	0.423**	0.367**	**0.799**	
PV	0.503**	0.414**	0.441**	0.248**	**0.825**

*p < 0.05, **p < 0.01. The bold number is the square root of AVE.

### Model-fitting test

The model fit was tested by analyzing the sample data by Amos Graphics software. All the indicators in Table 6 are within the acceptable range presented in Gefen’s study (29), indicating that the degree of fit of the model in this study is good.

**Table 6:** Model-fitting index.

Index	Evaluation criterion	Fits of this model	Adaptation judgment
CMIN/DF	<3	0.972	Match
GFI	≥0.9	0.963	Match
AGFI	[0.7–0.9]	0.945	Match
NFI	≥0.9	0.964	Match
RFI	≥0.9	0.954	Match
RMSEA	<0.10	0.074	Match

### Hypothesis test

This study conducted path analysis in AMOS. To demonstrate the results of the hypotheses, the standard pathway coefficient for the variables in each hypothesis was evaluated. A *p*-value less than 0.05 in the table indicates that the pathway is significant, and the assumption is supported. All hypotheses are supported (as shown in Table 7). PC (*β* = 0.268, *p* < 0.001), TA (*β* = 0.256, *p* < 0.001), and DS (*β* = 0.433, *p* < 0.001) had a direct and significant positive effect on DUI. TA (*β* = 0.331, *p* < 0.001) and PC (*β* = 0.335, *p* < 0.001) positively affected DS. The positive effect of PC on TA (*β* = 0.384, *p* < 0.001) was also significant.

**Table 7:** Results of hypothesis test.

Hypothesis	Pathway	*β*	S.E.	C.R.	*P*	Result
H1	DUI < ---PC	0.268	0.067	3.997	***	Support
H2	DS < ---PC	0.335	0.08	4.178	***	Support
H3	TA < ---PC	0.384	0.078	4.95	***	Support
H4	DUI < ---TA	0.256	0.066	3.882	***	Support
H5	DS < ---TA	0.331	0.078	4.225	***	Support
H6	DUI < ---DS	0.433	0.067	6.414	***	Support

*p < 0.05, **p < 0.01, ***p < 0.001.

### Mediating effect of technology anxiety and dissatisfaction

**Figure 2:**
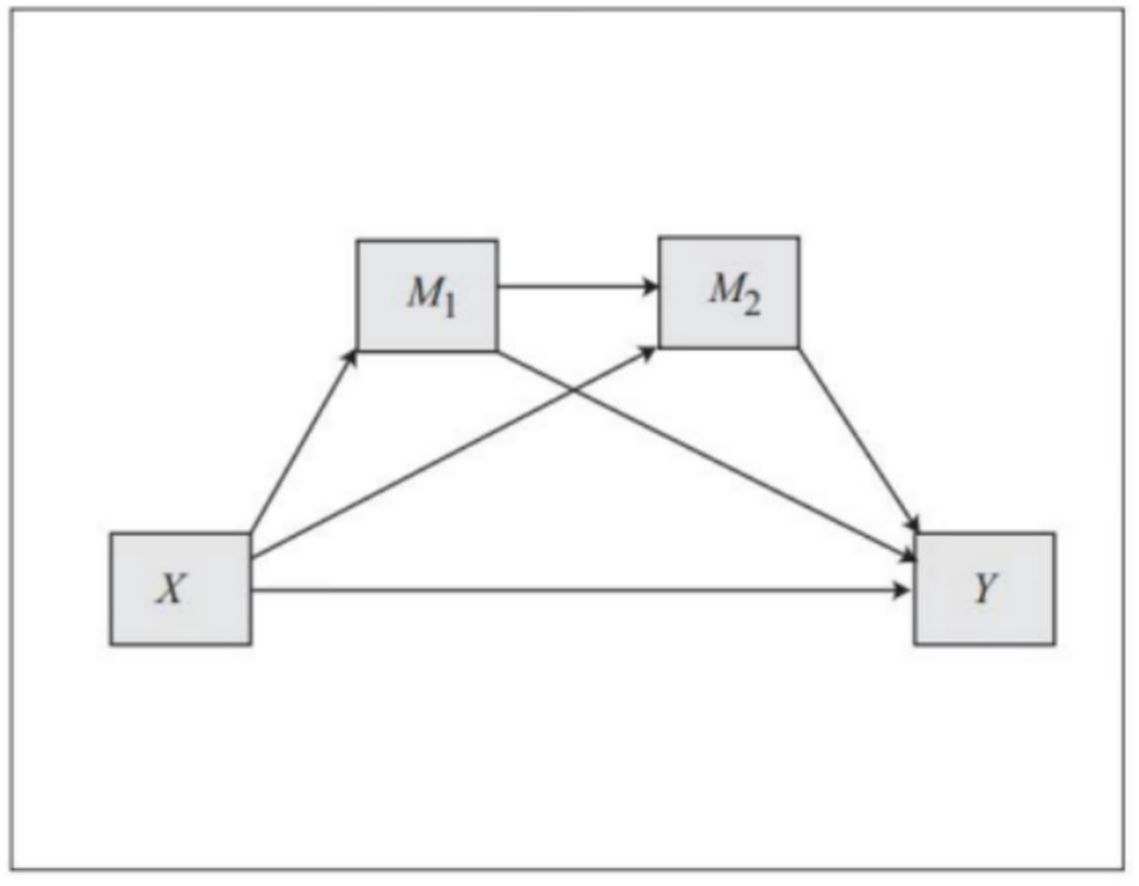
Model 6 in process (49).

This study conducted a test of the chain mediation effect in Process Model 6 (see Figure 2). In the research model, privacy concerns was the independent variable, with discontinuous usage intention as the dependent variable, and technology anxiety and dissatisfaction as chain mediating variables, which constitute a typical chain mediating model. The mediating effect was tested by Bootstrap sampling, and the measured data showed that there were total, total indirect and indirect significant effects (as shown in Table 8). The results are derived based on direct and indirect path coefficients, as shown in Table 9. In the indirect effect, the path “PC- > DS- > DUI” had the highest proportion of (24.76%) and the most significant effect 0.1040 (95%, CI = [0.0564, 0.1588]). The pathway “PC- > TA- > DUI” was also established, with the effect value of 0.0703 (95%, CI = [0.0334, 0.1116]). The effect value of the pathway “PC- > TA- > DS- > DUI” was 0.0313 (95%, CI = [0.0131, 0.0577]) (as shown in Table 9). The results indicate that privacy concerns of older adults’ will affect their dissatisfaction, thus indirectly affect their discontinuous usage intention of online paid health consultation platforms. Privacy concerns of the online paid health consultation platform will also cause their technology anxiety, which leads to discontinuous usage intention among older adults. Technology anxiety and dissatisfaction, as mediating factors, play a positive role in the relationship between privacy concerns and discontinuous usage intention.

**Table 8:** Chain mediation effect.

Latent variables	TA		DS		DUI	
	*β*	*t*	*β*	*t*	*β*	*t*
PC	0.32	5.32***	0.29	4.81***	0.21	4.62***
TA			0.27	4.55***	0.21	4.79***
DS					0.36	7.71***
R	0 0.31		0.45		0.67	
R^2^	0.10		0.20		0.45	
F	28.31***		32.17***		70.40***	

*p < 0.05, **p < 0.01, ***p < 0.001.

**Table 9:** Each path containing mediating action.

Pathway	Effect value	Standard error	95% confidence interval		Effect percentage
			Lower limit	Superior limit	
Total	0.4200	0.0504	0.3208	0.5192	100%
Direct	0.2144	0.0464	0.1231	0.3057	51.05%
Total indirect	0.2056	0.0365	0.1351	0.2799	48.95%
PC- > TA- > DUI	0.0703	0.0201	0.0334	0.1116	16.74%
PC- > DS- > DUI	0.1040	0.0260	0.0564	0.1588	24.76%
PC- > TA- > DS- > DUI	0.0313	0.0114	0.0131	0.0577	7.45%

### Moderating effect of price value

**Figure 3:**
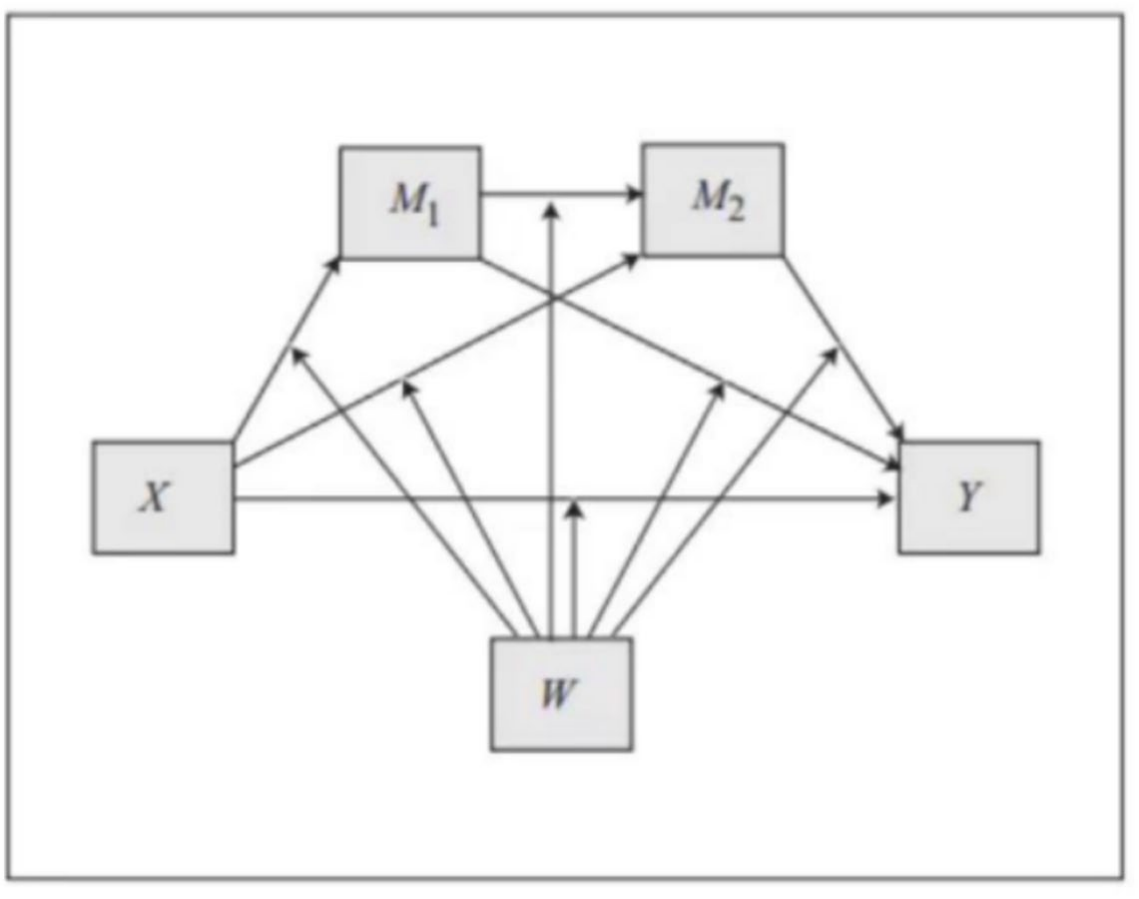
Model 92 in process.

The study used the Process of model 92 (see Figure 3) to analyze the moderating effects of the platform feature “PV” on PC, TA, DS, and DUI. First, we conducted an analysis of the moderating effects on the paths of direct influence, and the results were significant. Next, we analyzed the different chain-mediated paths separately, and the moderating effect of PV was different for the high and low groups. Results are shown in Tables 10, 11.

**Table 10:** Moderated mediating effect.

Latent variables	TA			DS			DUI		
	Coeff	SE	*t*	Coeff	SE	*t*	Coeff	SE	*t*
Constant	−0.02	0.09	−0.18	0.02	0.08	0.19	5.28	0.06	88.89**
PV	0.13	0.07	1.92	0.21	0.06	3.32**	0.17	0.05	3.56**
PC	0.28	0.07	4.30**	0.21	0.06	3.40**	0.16	0.05	3.53**
TA				0.24	0.06	4.29**	0.21	0.04	4.72**
DS							0.35	0.05	7.56**
PC *PV	0.02	0.04	0.59	−0.08	0.04	−2.09*	0.07	0.03	1.99**
TA *PV				0.10	0.04	2.21*	−0.14	0.03	−4.21**
DS *PV							−0.02	0.04	−0.56
*R* ^2^	0.11			0.27			0.52		
*F*	10.73			18.12			38.44		

*P < 0.05, **P < 0.01.

**Table 11:** Direct and indirect effects of PC on DUI with PV as moderating factor.

Pathway	Moderated mediation	Moderated mediation values	β	SE or (Boot SE)	BC 95% CI	
					*t* and/or (Boot Lower)	*p* and/or (Boot Upper)
PC- > DUI	PV	Low	0.07	0.07	1.11(−0.06)	0.27 (0.20)
		Media	0.16	0.05	**3.53 (0.07)**	**0.001 (0.25)**
		High	0.25	0.06	**4.04 (0.13)**	**0.0001 (0.37)**
PC- > TA- > DUI		Low	0.10	0.03	**(0.03)**	**(0.16)**
		Media	0.06	0.02	**(0.02)**	**(0.10)**
		High	0.01	0.03	(−0.04)	(0.06)
PC- > DS- > DUI		Low	0.12	0.03	**(0.05)**	**(0.19)**
		Media	0.07	0.02	**(0.03)**	**(0.12)**
		High	0.03	0.03	(−0.03)	(0.09)
PC- > TA- > DS- > DUI		Low	0.01	0.01	(−0.002)	(0.04)
		Media	0.02	0.01	**(0.01)**	**(0.05)**
		High	0.04	0.02	**(0.01)**	**(0.09)**

The effect of boot lower and boot upper is significant if they do not pass through zero, which has been bolded.

**Figure 4:**
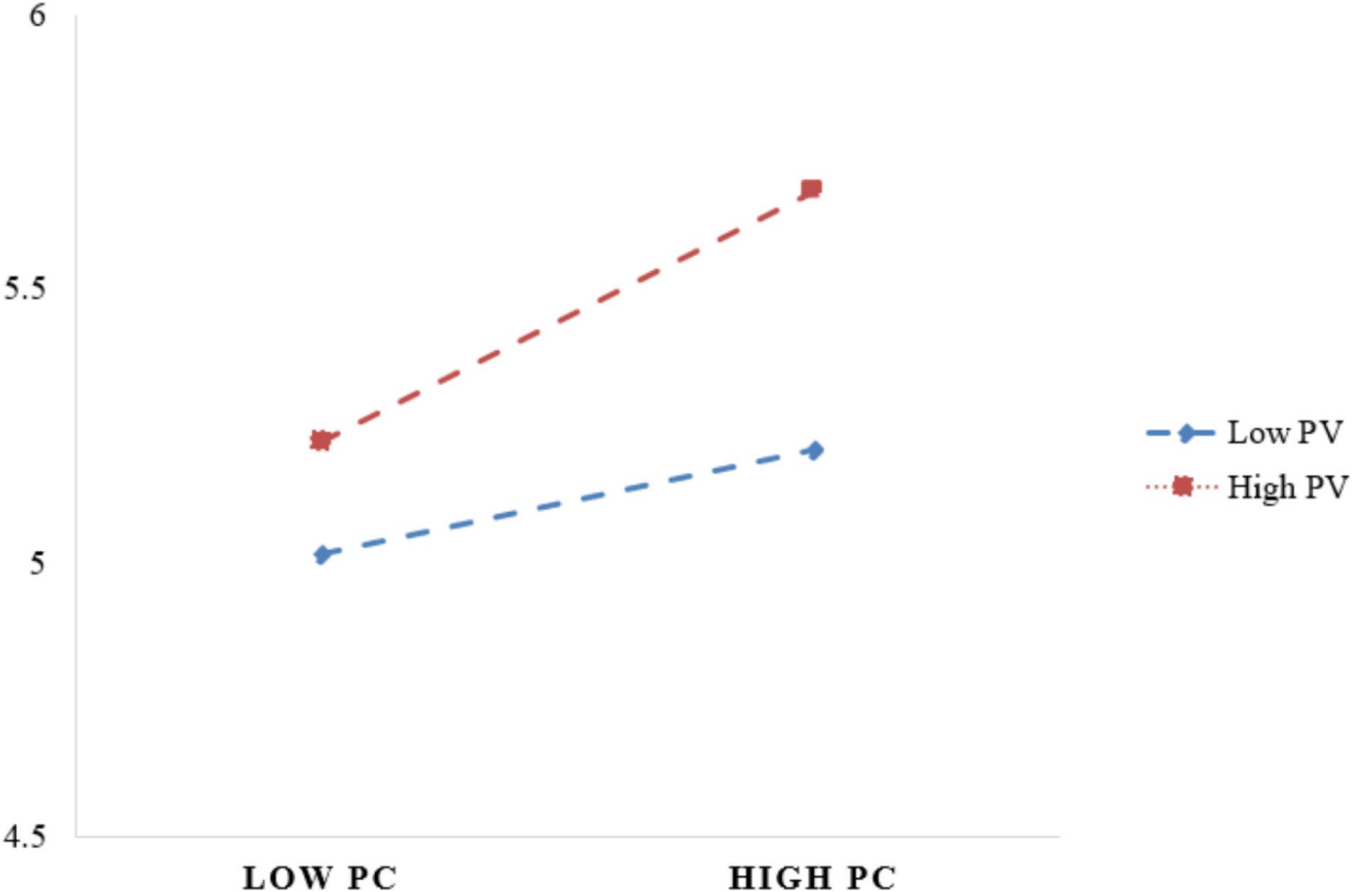
Moderation of price value on privacy concerns and discontinuous usage intention.

The moderation analysis indicates that PV significantly moderates the relationship between PC and DUI (*p* < 0.001). For the group with high PV, PC significantly increases the DUI (*β* = 0.25, SE = 0.06, *p* = 0.0001, LLCI = 0.13, ULCI = 0.37), whereas for the group with low PV, the effect of PC on DUI is not significant (*β* = 0.07, SE = 0.07, *p* = 0.27, LLCI = −0.06, ULCI = 0.20). To describe this interaction effect, we plot the DUI of PC predictions separately for low versus high levels of PV (as shown in Figure 4). Simple slope tests indicated that higher levels of PC were associated with higher DUI for high PV individuals (*β*simple = 0.25, *p* < 0.001). However, for those with lower PV levels, the association between lower levels of PC and DUI was not significant (*β*simple = 0.07, *p* > 0.05). Therefore, H7 was partly supported.

The PV also had some moderating role in the chain mediating effect. On the pathway “PC- > TA- > DUI,” the low PV (*β* = 0.10, Boot SE = 0.03, LLCI = 0.03, ULCI = 0.16) was significant because zero was not included in the CI result, but it was not significant in high PV (*β* = 0.01, Boot SE = 0.03, LLCI = −0.04, ULCI = 0.06).

On the pathway “PC- > DS- > DUI,” low PV (*β* = 0.12, Boot SE = 0.03, LLCI = 0.05, ULCI = 0.19) had a significant moderating effect, while high PV (*β* = 0.03, Boot SE = 0.03, LLCI = −0.03, ULCI = 0.09) had an insignificant moderating effect.

On the pathway “PC- > TA- > DS- > DUI,” high PV (*β* = 0.04; Boot SE = 0.02; LLCI = 0.01; ULCI = 0.09) had a significant moderating effect, while low PV (*β* = 0.01; Boot SE = 0.01; LLCI = −0.002; ULCI = 0.04) had an insignificant moderating effect.

In summary, H8, H9, and H10 are partly supported.

## Discussion

With the improvement of living standards and quality, consumers pay more attention to their health, and the impact of living habits on health has been widely recognized. Online paid health consultation platforms can provide people with efficient and convenient health consultation services. Especially for older adults group with more health inquiry needs (27), smart medical care can help them solve their physiological disadvantages such as travel difficulties. However, during the use of digital health consulting platforms, older adults also face problems, such as not being able to operate and unable to see the interface clearly, which will further lead to negative emotions such as technology anxiety, dissatisfaction, and unwillingness to continue to use among older adults. As a result, older adults are likely not to continue to use online health paid consulting platforms. We studied the relationship among privacy concerns, technology anxiety, dissatisfaction and discontinuous usage intention among older adults when using online paid health consultation platforms. We found that privacy concerns, technology anxiety, and dissatisfaction directly affect the discontinuous usage intention of online health consultation platforms, among which technology anxiety and dissatisfaction play a mediating role. Price value moderates the impact of privacy concerns on older adults’ discontinuous usage intention.

### Theoretical implication

In this study, privacy concerns directly affect dissatisfaction and discontinuous usage intention, which is consistent with previous studies (19, 23). Scholars believe that personal concerns about health data privacy can affect their willingness to use the technology and their physical and mental health (12, 20). This indicates that when individuals are concerned about the privacy and security of their personal information, they are more likely to show discontinuous usage intention, i.e., to abandon, or to reduce the use of a certain product, service or platform. This may be because individuals fear that their information may be abused, exposed, or unauthorized access, affecting their privacy rights and security. Due to the low literacy, older people have stronger privacy concerns, which causes their unsatisfactory feelings and discontinuous usage intention (25, 26, 35).

Our results indicated that technology anxiety positively affected the dissatisfaction and discontinuous usage intention of online paid health consultation platforms, consistent with the conclusions of some previous studies. Previous studies have suggested that technology anxiety is one of the main barriers to the use of new technology platforms or technology services (9). High-technology anxiety has become a prominent feature of older users. Older people in developing countries experience more obvious technology anxiety when using mobile health technology, compared with those in developed countries (36). Older users’ technical anxiety affects their use of various aspects of information technology services and products, such as smart devices (10), health information acquisition (35), and mobile health services (8). In some studies, technical anxiety has little impact on users’ intention to use. The reason may be that respondents are users with much experience in Internet use, who are not afraid to use new technologies. With the improvement of experience level, their negative impact may decrease (23, 24).

Privacy concerns in older adults in our study positively affected the psychological state of technical anxiety. This finding is not entirely consistent with the findings of previous studies (46, 47). A previous study (37) suggested that users’ concern for information privacy moderates the relationship between computer anxiety and behavioral intentions, while another study (32) proved that technology anxiety did not have an impact on privacy concerns. Previous studies showed that older adults were less likely to use information technology than young people were (7). In our study, the influence of privacy concerns on technical anxiety may originates from the SSO model, where we define the privacy concerns of older people as their stressors, while technical anxiety is a psychological burden and discontinuous usage intention as a result. Older users often worry that using the Internet possibly result in personal information exposure, leading to the safety of their property (38). Meanwhile, older adults may encounter operational problems when using online platforms, which is more likely to cause the accidental disclosure of personal information, prone to technical anxiety and thus resistance to using online platforms.

Price value’s moderating role in older people’s discontinuous usage intention is another difference between our study and previous studies. We combine the characteristics of the online paid health consultation platforms with the sensitivity of older adults to the price, and select value price as the moderating variable. As indicated in our study, price value has a certain impact on older adults’ discontinuous usage intention of online paid health consultation platforms, but the overall moderating effect is not significant. This is partly consistent with the research hypothesis, which may be affected by the measurement of variables. The participants are older adults who can fill out the electronic questionnaire. This group may have a higher economic level, so their value price sensitivity will be higher than the average level. Consumers often compare the costs they spend with the benefits they get from them while using technical services (39). Previous studies suggested that price value significantly and positively affected the willingness to behave (15, 16). Consumers will increase their use of technology services when they get more benefits (40) when using online health platforms (14).

To sum up, it can be seen that our study not only innovatively puts forward the theoretical model of older adults’ discontinuous usage intention of online paid health consulting platforms, but also enriches the influencing factors of older adults’ discontinuous usage intention to use information technology. Our research model combines the characteristics of both online paid health consulting platforms and older adults, which are rarely considered in previous studies, and adds the factors including price value, technology anxiety, and privacy concerns to the discontinuous usage intention model. In particular, we investigated the mediating role of technology anxiety and the moderating role of price value, which were not considered in previous studies. Our study may make a theoretical contribution to the current research on information adoption. In terms of health information search for older adults, the online paid health consulting platform is a different context. Future research can be conducted in other contexts to further explore the credibility and feasibility of our research model.

### Practical implication

Based on the conclusions, this study has the following practical implications. For families, more technological assistance can alleviate the technological anxiety of older adults and help them improve their level of technology use. In terms of platform design, to address the privacy concerns and technological anxiety of older adults, privacy protection features (such as anonymous consultation) can be enhanced, and the user interface can be simplified to improve the older users’ experience. In addition, platforms should adjust their pricing strategies, introducing tiered pricing or family-sharing packages to reduce the price sensitivity of older adults, while also offering free trial opportunities. For society, it is necessary to create technological trust atmosphere, actively providing technological guidance and training for older adults, clarifying the positive significance of platforms, and enhancing the technological confidence and trust of older adults. Governments and enterprises should also cooperate with each other to assist in optimizing services with age-friendly digital health policies.

### Limitations and future research

The limitations of this study are as follows. The research objects are Chinese older adults, thus our findings may have certain limitations and cannot represent the whole older adults group well. The future research can be jointly carried out in many countries and regions to expand the sample size. The effect of positive emotions on discontinuous usage intention among older adults is not taken into account in our model, which could be considered in the future studies. The control variables can be supplemented to the research model in future studies, by adding demographic characteristics including income and gender, to enrich the dimensions of the study.

## Conclusion

This study focuses on the influencing factors of discontinuous usage intention of online paid health consultation platforms. Based on personal, platform, and service factors, privacy concerns, technology anxiety, dissatisfaction, and price value are summarized as influencing factors. The results show that privacy concerns, technology anxiety, and dissatisfaction affect older adults’ discontinuous usage intention, and price value plays a moderating role. Among them, the mediating effect of moderating is different from previous studies. As the current aging population intensifies and the global economy is booming, we could not ignore the power of older adults on social development, and their pursuit of high personal living standards will also promote the emergence and upgrading of new industries. In particular, in the field of healthcare, older adults have a high desire for a healthy life. Therefore, in the development of online medical technology, the actual needs of older adults and their characteristics should be considered to alleviate the problem result from aging.

## Data Availability

The datasets presented in this study can be found in online repositories. The names of the repository/repositories and accession number(s) can be found in the article/supplementary material.
